# Beyond the Lumen: Molecular Imaging to Unmask Vulnerable Coronary Plaques

**DOI:** 10.3390/jcdd12020051

**Published:** 2025-01-30

**Authors:** Geoffrey Currie, Hosen Kiat

**Affiliations:** 1 School of Dentistry and Medical Sciences, Charles Sturt University, Wagga Wagga, NSW 2678, Australia; hosen.kiat@chi.sydney; 2 College of Health and Medicine, Australian National University, Canberra, ACT 2601, Australia; 3 Faculty of Medicine, Health and Human Sciences, Macquarie University, Sydney, NSW 2109, Australia

**Keywords:** vulnerable plaque, unstable plaque, plaque rupture, molecular imaging, positron emission tomography

## Abstract

Vulnerable coronary atherosclerotic plaque involves a dynamic pathophysiologic process within and surrounding an atheromatous plaque in coronary artery intima. The process drastically increases the risk of plaque rupture and is clinically responsible for most cases of acute coronary syndromes, myocardial infarctions, and sudden cardiac deaths. Early detection of vulnerable plaque is crucial for clinicians to implement appropriate risk-mitigation treatment strategies, offer timely interventions, and prevent potentially life-threatening events. There is an imperative clinical need to develop practical diagnostic pathways that utilize non-invasive means to risk-stratify symptomatic patients. Since the early 1990s, the identification of vulnerable plaque in clinical practice has primarily relied on invasive imaging techniques. In the last two decades, CT coronary angiogram (CTCA) has rapidly evolved into the prevalent non-invasive diagnostic modality for assessing coronary anatomy. There are now validated plaque appearances on CTCA correlating with plaque vulnerability. It is worth noting that in clinical practice, most CTCA reports omit mention of vulnerable plaque details because spatial resolution (0.3–0.5 mm) is often insufficient to reliably detect some crucial features of vulnerable plaques, such as thin fibrous caps. Additionally, accurately identifying vulnerable plaque features requires substantial expertise and time, which many cardiologists or radiologists may lack in routine reporting. Cardiac magnetic resonance imaging (cMRI) is also non-invasive and allows simultaneous anatomic and functional assessment of coronary plaques. Despite several decades of research and development, routine clinical application of cMRI in coronary plaque imaging remains hampered by complex imaging protocols, inconsistent image quality, and cost. Molecular imaging with radiotracers, specifically positron emission tomography (PET) with sodium fluoride (Na^18^F PET), have demonstrated significant potential as a sensitive and specific imaging procedure for diagnosing vulnerable coronary artery plaque. The study protocol is robust and brief, requiring minimal patient preparation. Compared to CTCA and cMRI, the diagnostic accuracy of this test is less dependent on the experience and expertise of the readers. Furthermore, validated automated quantitative algorithms complement the visual interpretation of the study, enhancing confidence in the diagnosis. This combination of factors makes Na^18^F PET a promising tool in cardiology for identifying high-risk coronary plaques.

## 1. Introduction

Coronary atherosclerosis, clinically denoted as coronary artery disease (CAD), is a chronic, progressive inflammatory disease characterized by the accumulation of cholesterol plaque in the arterial walls [1,2]. In clinical practice to date, diagnostic imaging for CAD assessment aims to determine its hemodynamic significance, classifying it as obstructive or non-obstructive. This evaluation has been achieved through various methods that can be broadly categorized into anatomical and functional assessments. Anatomical assessment predominantly focuses on the physical characteristics of coronary arteries and includes techniques such as coronary angiography by cardiac catheterization and computed tomography (CT) coronary angiography (CTCA). Functional assessment evaluates the physiological impact of stenoses on myocardial blood flow and includes stress myocardial perfusion studies and stress echocardiograms, which assess CAD by detecting inducible ischemia. While these methods have been widely adopted, it is worth noting that cardiac magnetic resonance imaging (MRI) has shown promise in CAD assessment. However, its utilization has remained limited to a few tertiary centers, primarily due to cumbersome imaging protocols, cost, availability, inconsistency in imaging quality, and the expertise required for interpretation.

**Figure 1:**
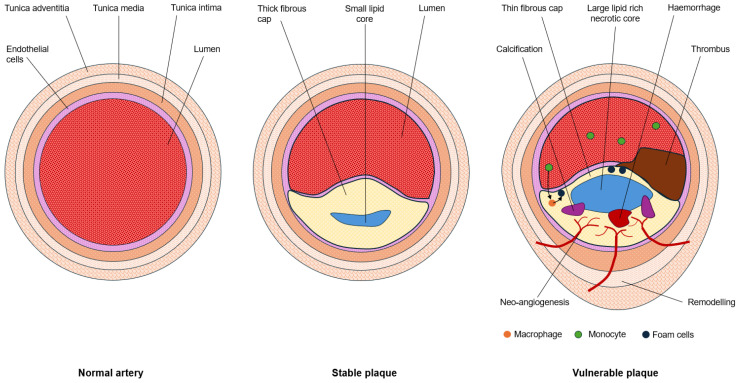
Cross section through an artery schematically representing a normal vessel (**left**), a stable plaque with a small lipid core and thick fibrous cap (**middle**), and a vulnerable plaque with monocyte migration, macrophage deterioration, micro-calcification, neo-angiogenesis, large lipid-rich necrotic core, thin fibrous cap, intra-plaque hemorrhage, thrombus formation, and vessel wall remodeling (**right**).

These stable, obstructive-type atherosclerotic plaques tend to cause ischemia (2). Unstable atherosclerotic plaque is more susceptible to erosion or rupture and tends to cause occlusion and infarction [2]. Plaque vulnerability is a life-threatening risk, and the ability of imaging to differentiate stable from vulnerable plaque remains a challenge [1,2]. In recent years, landmark clinical trials such as ISCHEMIA, BARI-2D, and REVIVED-BCIS2 [3,4] have provided compelling evidence that most revascularization procedures using coronary percutaneous intervention (PCI) do not offer significant prognostic benefits for patients with stable CAD. These findings challenge the long-held belief that mechanical intervention is always beneficial in managing stable CAD. The lack of demonstrable benefits from these interventions highlights two crucial points. First, it suggests that stable atherosclerotic plaques may not pose an immediate threat to patients, contrary to previous assumptions. This revelation implies that stable plaques are clinically benign in many cases. Second, these trials underscore the importance of developing methods to identify unstable or vulnerable plaques prone to rupture and potentially trigger acute cardiac events. This paradigm shift in understanding emphasizes the need for a more nuanced approach to CAD management, focusing on accurate risk stratification and targeted interventions for truly high-risk patients. The medical community now faces the challenge of refining diagnostic strategies to distinguish between stable and unstable plaques effectively. Such advancements could revolutionize the prevention and treatment of acute coronary syndromes, leading to more personalized and efficient care for patients with CAD. New imaging probes require careful consideration of the pathogenesis of plaque evolution and the molecular hallmarks of plaque vulnerability (Figure 1).

The aim of this manuscript is to provide an insight the molecular hallmarks of plaque vulnerability and to provide an overview of molecular imaging probes suitable for imaging. A deeper commentary of the widely documented ^18^F FDG PET in inflammation associated with high-risk plaque will be briefly expanded. A systematic review is also provided examining more critically and thoroughly the emerging application of ^18^F NaF in plaque vulnerability with the purpose of providing an evidence-based foundation for its use. 

### 1.1. Hallmarks of Plaque Vulnerability 

**Figure 2:**
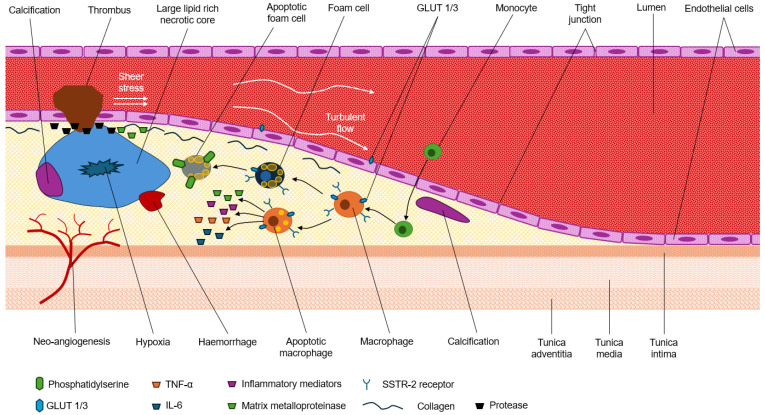
Schematic representation of the hallmarks of vulnerable plaque.

Vulnerable plaques are atheroma susceptible to rupture [1]. Consequently, vulnerable plaques are implicated in acute cardiovascular events [1,5]. Importantly, plaque size or lumen encroachment are not predictors of plaque vulnerability in the coronary arteries [6]. There are a number of morphological features and molecular behaviors typical of vulnerable plaque that could be utilized for risk assessment and classification of atherosclerotic plaques, and importantly, differentiation from stable phenotypes Figure 2. Fundamentally, plaque vulnerability emerges from the complex and dynamic interplay between a growing necrotic core, thinning fibrous cap, and sheer stress in coronary lumen [1,2,7]. 

A thinning fibrous cap leaves the plaque susceptible to rupture and thrombus formation [1,7], especially with increased sheer stress and turbulent blood flow associated with lumen narrowing. The thinning fibrous cap is inclusive of the connective tissue extracellular matrix, which is composed of, among other complexes, collagen, elastic fibers, and proteoglycans [6]. As depicted in Figure 2, macrophages are accumulated in the plaque, which release inflammatory mediators. Indeed, atherosclerosis is an inflammatory process [2]. A higher degree of macrophage recruitment increases pro-inflammatory properties and causes fibrous cap thinning. Additionally, release of matrix metalloproteinase (MMP) from degenerated macrophages can hydrolyze and break down the matrix structure of the fibrous cap [1]. This damage destabilizes the fibrous cap and potentiates plaque vulnerability to rupture. Protease and MMP reduce collagen in the fibrous cap, which is further complicated by changes to tight junction resistance [1]. The net effect is increased permeability of the plaque, thinning of the fibrous cap, and increase risk of plaque rupture.

Plaque vulnerability and fibrous cap thinning are also driven by a growing lipid-rich necrotic core [1,7]. The necrotic core comprises cellular debris (e.g., products of phagocytosis), apoptotic macrophages, degraded foam cells, cholesterol/low-density lipoproteins (LDL), and micro-calcifications. Plaque vulnerability is lower where macrophage apoptosis inhibits foam cell formation but, in a lipid-rich micro-environment, increased apoptosis produces a high volume of remnants to drive both necrosis and necrotic core enlargement [1]. The plaque volume is also increased by micro-calcifications, which can accumulate and degrade the matrix and accumulate and enlarge the necrotic core; both actions produce fibrous cap thinning and susceptibility to rupture [1,7]. Intra-plaque hemorrhage can lead to a rapid increase in plaque volume and trigger a cascade of events. This process begins with the expansion of the necrotic core, which accumulates cellular debris including iron, cholesterol, and hemoglobin [1]. The presence of these components subsequently induces inflammation within the plaque. Moreover, the hemorrhage itself can result in oxidative stress, further contributing to plaque instability. This complex interplay of factors accelerates plaque progression and increases the risk of rupture [1]. 

A number of other factors contribute to plaque vulnerability. Coronary artery calcification (CAC) assessed by CT coronary calcium detection and quantification algorithms (CT-CAC) is not effective for identifying vulnerable plaque. Specific patterns of calcification, however, may offer a molecular-level insight into plaque vulnerability. Spotty calcification, for example, reflects macrocalcification and is more consistent with myocardial infarction risk than stable plaque [2]. Neo-angiogenesis results in new blood vessels into the base of the plaque that increase inflammation, increase the risk of hemorrhage, and increase plaque vulnerability to rupture [1]. Endothelial cell activation and expression of vascular cell adhesion protein 1 (VCAM-1) and plaque hypoxia have also been investigated as hallmarks of plaque vulnerability [1]. 

### 1.2. Imaging Plaque Morphology 

In clinical practice, non-invasive echocardiography and myocardial perfusion assessment of the hemodynamic significance of atherosclerotic CAD continue to be effective in the diagnosis of and prognostic assessment of CAD [2]. For assessment of coronary artery anatomy and identification of arterial lumen obstruction, cardiac catheterization coronary angiogram (CATH) remains the gold standard. Over the past three decades, CTCA has rapidly evolved, been validated, and widely adopted as a noninvasive diagnostic alternative to CATH. It has proven effective in reducing the number of CATH procedures performed in patients with low-intermediate to intermediate risk. CTCA plays an important role in cardiac risk stratification over and above traditional risk indices. For example, in the SCOT–HEART trial, CTCA-based changes in management reduced the myocardial infarction rate over 5 years of follow-up from 3.9% to 2.3% [8]. Despite differentiating obstructive from nonobstructive plaques, CTCA does not change mortality nor identify vulnerable plaques [2]. 

Atherosclerotic burden in the coronary arteries provides a prognostic indicator and can be evaluated using CT-CAC [2]. Cardiac event rates are virtually zero when CT-CAC detected absence of coronary artery calcium deposits. Twelve-year survival rates closely correlate with coronary artery calcium burden with a 99.4% survival for CAC Agaston quantitative score of 0–10 (relative risk 1.48), 97.8% survival for CAC score 11–100 (RR 3.61), 94.5% survival for CAC of 101–400 (RR 3.84), 93.0% survival for CAC of 401 to 1000 (RR 5.78–6.47), and 76.9% survival for CAC scores over 1000 (RR 9.36) [9]. Despite the prognostic value of CT-CAC, it remains a poor predictor of plaque vulnerability. Cardiac event rates were reported as higher for non-calcified versus calcified plaques (22.7% versus 5.5%) [10]. In another study, CTCA determined that “culprit” lesions had 92% more non-calcified plaque volume than non-culprit plaques [11]. 

Magnetic resonance imaging (MRI) offers valuable insights into plaque composition at a fundamental level. For instance, calcification can be distinguished from other plaque components by its hypointense T1 signal and significantly hypointense T2 and proton density (PD) signals [6]. In contrast, lipids exhibit marked hyperintensity on T1, hypointensity on T2, and hyperintensity on PD. Fibrous tissue typically appears isointense or slightly hyperintense across T1, T2, and PD sequences [6]. Thrombi can present with varying signal intensities on T1, T2, and PD [6]. However, MRI and MRI angiography face limitations in visualizing coronary arteries due to challenges such as spatial resolution constraints, cardiac motion, the presence of small vessels, non-linear tracking, and interference from adjacent myocardium [6]. 

These limitations hinder the routine clinical application of MRI for CAD diagnosis. Although MRI can differentiate major components of atherosclerotic plaques, including fibrous caps, lipid cores, calcium deposits, and hemorrhages, it is often outperformed by other imaging modalities like CTCA and invasive coronary angiography due to its lower spatial resolution and longer acquisition times [6]. Consequently, while MRI provides essential information about plaque composition and morphology without exposing patients to ionizing radiation or contrast agents, its practical use in assessing coronary artery disease remains limited.

Physiological imaging of plaque evolution is achievable with radionuclides. However, clinical utility in stratifying plaque vulnerability and rupture risk is limited (Table 1). For instance, during the early stages of fatty streak development in the arterial wall, radionuclides such as ^99m^Tc, ^111^In, ^123^I, ^18^F, and ^68^Ga, labelled LDL, can effectively identify localization within plaques, macrophages, and foam cells [12,13]. At this stage of plaque development, there is an increased expression of endothelial adhesion molecules like VCAM-1 (vascular cell adhesion molecule-1), which can be targeted using ^18^F-4V [1,12,13]. A significant challenge arises from non-specific accumulation due to the broad expression of VCAM-1 across various tissues, including those involved in inflammation.

Similarly, P-selectin, expressed by leukocytes, can be targeted with ^68^Ga fucoidan to assess macrophage density within plaques [12,13]. This primarily reflects plaque activity or growth rather than vulnerability. The primary radiotracer for evaluating inflammation is ^18^F FDG, although an alternative is the uptake of ^67^Ga citrate in macrophage-rich plaques [13]. Overall, while radionuclide imaging can provide insights into the physiological changes associated with plaque evolution, its effectiveness in predicting clinical outcomes remains constrained by issues such as specificity and the complexity of plaque biology.

### 1.3. Molecular Imaging of the Hallmarks of Plaque Vulnerability 

General nuclear medicine imaging, including single photon emission computed tomography (SPECT), shows promise with various radiopharmaceuticals for assessing plaque vulnerability, such as ^99m^Tc Annexin V for apoptosis. Unfortunately, its application is significantly hindered by several factors: poor spatial resolution, cardiac motion, and patient attenuation. Notably, the SPECT tracers that successfully identify vulnerable plaques are more effective in carotid imaging than in coronary imaging.

In contrast, molecular imaging with positron emission tomography (PET) offers several advantages for evaluating plaque vulnerability. The integration of anatomical imaging with CT (PET/CT), whether through CTCA or CAC scoring and the implementation of cardiac motion correction, significantly improves both resolution and image quality [2]. Additionally, blood pool correction algorithms and delayed imaging using longer-lived radionuclides, such as ^64^Cu, can be beneficial. The higher count sensitivity of long-bore PET scanners, including total-body PET, also enhances imaging capabilities.

Imaging the coronary arteries presents unique challenges compared to carotid arteries due to their smaller size, deeper location within the body, and proximity to the myocardium, which may accumulate radionuclides. Moreover, radiopharmaceuticals often exhibit high blood activity that complicates the differentiation from plaque accumulation. For instance, ^18^F FDG demonstrates a significant and variable degree of blood pool activity at 60 min post-intravenous administration alongside myocardial accumulation. Similarly, ^68^Ga DOTATATE can show non-specific accumulation in myocardium and prolonged retention in the vascular pool. In contrast, ^18^F NaF is quickly extracted from the vascular pool and exhibits negligible uptake in myocardium. Substituting ^68^Ga DOTATATE (with a half-life of 68 min) for ^64^Cu SarTATE (which has a half-life of 12.7 h) alters patient dosimetry while allowing for later imaging time points that enhance the target-to-background ratio. Furthermore, the high-count sensitivity of long axial field-of-view PET (total-body PET) may facilitate delayed imaging of shorter-lived radionuclides like ^68^Ga and ^18^F, thereby improving the target-to-background ratio.

Macrophages can be effectively targeted for imaging through receptor imaging techniques. Notably, macrophages exhibit a high expression of somatostatin 2 receptors (SSTR2), which can be specifically targeted using radiopharmaceuticals such as ^68^Ga DOTATATE or ^64^Cu SarTATE [14]. Additionally, the expression of mannose receptors can be targeted using ^18^F fluoro-D-mannose, while folic acid receptors can be imaged with ^18^F fluorofolic acid [12,13,14]. Chemokine receptors may also be targeted using ^68^Ga pentixafor [12,13,14]. Furthermore, macrophage proliferation leads to increased glycolysis, making ^18^F FDG a promising radiopharmaceutical that accumulates in areas with elevated macrophage presence [1,12,13,14]. On a more fundamental level, monocyte migration can be evaluated using leukocytes labelled with either ^111^In or ^99m^Tc [12,13]. Macrophages also express GLUT-1 and GLUT-3, facilitating glucose uptake as an energy source, particularly in hypoxic conditions [12]. Hypoxia, resulting from inadequate perfusion to the necrotic core, serves as an indicator of plaque vulnerability. However, imaging of coronary arteries using ^18^F FDG presents challenges due to adjacent myocardial uptake and tracer retention in the vascular space, complicating the differentiation of small areas of plaque accumulation [14,15]. The activity or density of macrophages can also be assessed using radiotracers like ^11^C choline or ^18^F fluorocholine, which are incorporated into the lipid components of activated macrophage cell membranes [14]. Among the various imaging modalities, ^18^F-FMISO PET/CT is widely used for hypoxia imaging, although alternatives such as ^64^Cu-ATSM PET/CT and PET/MRI have also been explored [12,13,14].

Pro-inflammatory cells, particularly T-lymphocytes, are significant markers of plaque vulnerability [12]. Activated T-lymphocytes overexpress interleukin-2 (IL-2), providing a highly active marker for assessing plaque vulnerability [12,13,14]. Currently, the capability limitations of SPECT limit the application of IL-2 tracers like ^99m^Tc-HYNIC-IL-2. In contrast, neo-vascularization or angiogenesis serves as a potent marker for plaque vulnerability and is accessible for imaging with PET radiopharmaceuticals such as ^18^F galacto-RGD and ^68^Ga-NOTA-PRGD2 [12,13,14]. Specifically, newly formed blood vessels in plaques can be identified through integrin α_v_β_3_ expression on vascular cells [1,12,13,14]. Matrix metalloproteinases (MMPs) released from macrophages contribute to the degradation of the fibrous cap of plaques, increasing their susceptibility to rupture [12,13,14]. Various radiotracers have been developed to evaluate MMP activity, such as those labelled with either ^99m^Tc or ^111^In, although clinical success has been limited. Recent studies have focused on the role of radiotracers like ^18^F or ^68^Ga FAPI due to their ability to accumulate in thinner fibrous caps where collagen breakdown occurs [14].

**Figure 3:**
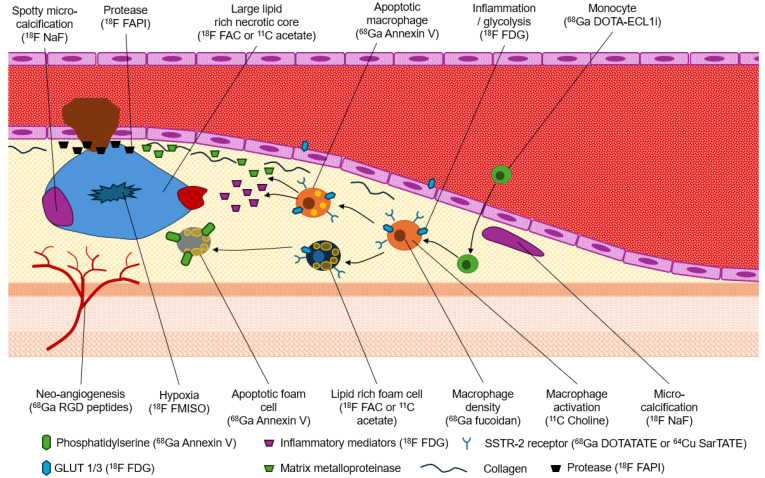
Schematic representation of the key hallmarks of vulnerable plaque that offer molecular imaging targets.

Apoptosis in macrophages and foam cells within the necrotic core serves as another marker for plaque vulnerability and can be imaged utilizing phosphatidylserine-targeted radiotracers [1,12,14]. Historically, apoptosis was assessed using ^99m^Tc-Annexin V. However, this approach faced limitations associated with SPECT imaging. More recently developed ^68^Ga-Annexin V has shown promise for PET/CT imaging of apoptosis in vulnerable plaques [12]. Importantly, ^68^Ga-Annexin V also accumulates in hydroxyapatite within matrix vesicles since micro-calcification is triggered by cell death [12]. Additionally, ^18^F NaF binds to hydroxyapatite as a marker for micro-calcifications and plaque vulnerability, particularly in areas exhibiting active micro-calcification [13,14]. The advantage of ^18^F NaF PET/CT lies in its preferential accumulation in micro-calcifications that are less likely to yield positive results on CAC compared to macrocalcifications associated with CAC positivity [15]. Despite this advantage, calcification may correlate with plaque stability [16]. A summary of molecular imaging targets for vulnerable plaques is illustrated in Table 2 and Figure 3.

### 1.4. Inflammation

Inflammation plays a crucial role in atherosclerosis, as it may help predict high-risk plaques that are vulnerable to rupture. The radiotracer ^18^F FDG accumulates in cells that rely on glucose for their metabolic activity, including macrophages and lymphocytes [16]. A significant body of literature has examined the use of ^18^F FDG PET/CT imaging in identifying vulnerable plaques, with most studies confirming its effectiveness as a marker of macrophage density [16]. 

There are limitations associated with non-specific ^18^F FDG accumulation in the myocardium, blood, and other inflammatory tissues. A more promising imaging target is the upregulation or overexpression of SSTR2 on macrophages, which enables PET/CT imaging using ^68^Ga DOTATATE or ^64^Cu SarTATE. Notably, ^68^Ga DOTATATE imaging of macrophage density as an inflammation marker shows a strong correlation with histological and gene expression analyses [16]. 

**Figure 4:**
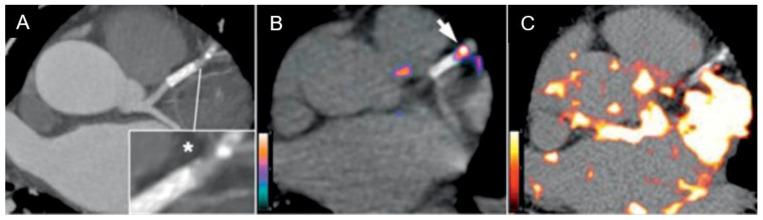
Comparison between CTCA with culprit stenosis (star in the pop out box) in the left anterior descending artery (**A**), ^68^Ga DOTATATE showing localized accumulation (arrow) corresponding to macrophage density and inflammation (**B**), and ^18^F FDG with more subtle focal accumulation confounded by high background accumulation (**C**). Adapted (cropped) from Tarkin et al. [17]. 2017 Copyright the Authors.

**Figure 5:**
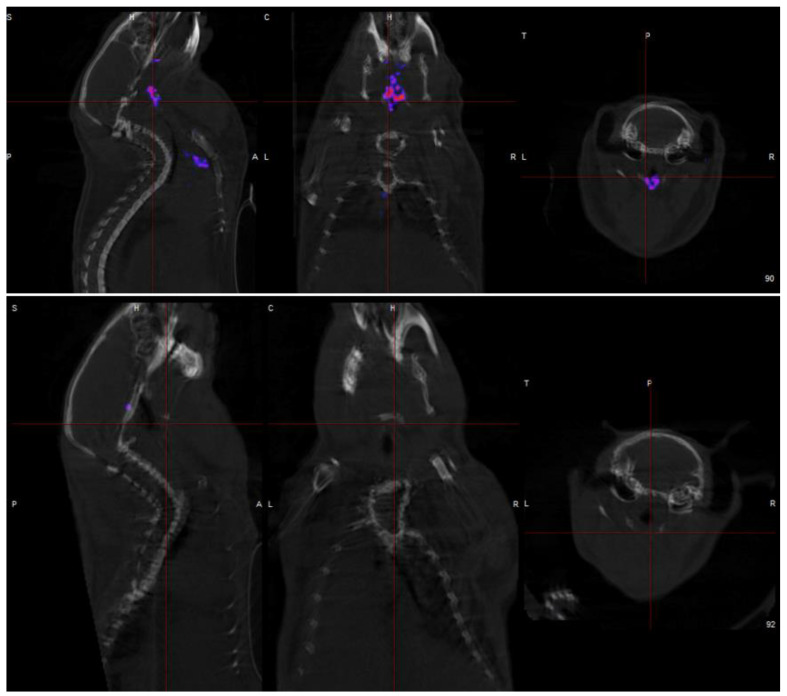
Representative murine model showing intense ^64^Cu SarTATE accumulation at the site of unstable plaque (**top**) and absence of ^64^Cu SarTATE accumulation in stable plaque (**bottom**). A—anterior; P—posterior; L—left; R—right; H—Head; C—Coronal; T—Transverse; S—Sagittal.

The comparative performances of ^18^F FDG and ^68^Ga DOTATATE in assessing vulnerable plaques are illustrated in Figure 4. Due to the short half-life of ^68^Ga, imaging should occur approximately 60 min after radiopharmaceutical administration when blood pool levels remain high. Delayed imaging using the longer half-life radiotracer ^64^Cu can enhance the target (plaque) to background (blood) ratios, as shown in Figure 5

### 1.5. Micro-Calcifications

**Figure 6:**
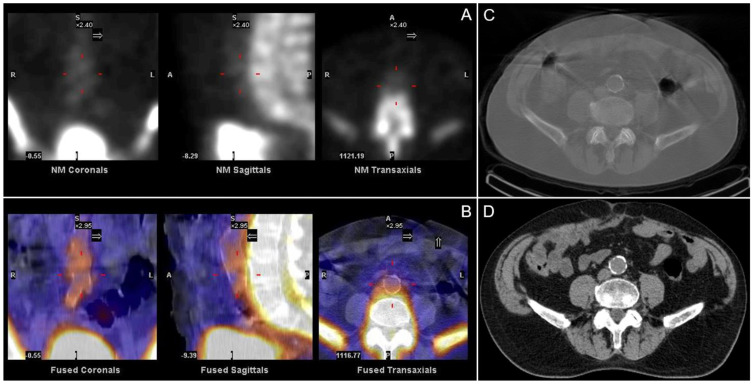
SPECT/ (**A**) and co-registered SPECT/CT (**B**) to the non-anatomical CT (**C**) of the spine using ^99m^Tc MDP shows typical blurring of the anterior vertebral body that corresponds to calcification of the descending aorta on the CT (**D**).

Imaging micro-calcifications has been a long-standing practice in nuclear medicine. The application of ^99m^Tc PYP for visualizing myocardial infarction is based on the localization of this phosphate radiopharmaceutical in the hydroxyapatite of micro-calcifications found in infarcted tissue. This principle extends to various conditions where calcium or phosphate analogues accumulate, such as in electrical burns illustrated by bone scans performed on patients post-defibrillation, soft tissue tumors like lung carcinoma, scar tissue from injections or surgical sites, and soft tissue injuries such as contusions. Furthermore, atherosclerotic micro-calcifications can occasionally be detected on a ^99m^Tc MDP (or HDP) bone scan Figure 6.

**Figure 7:**
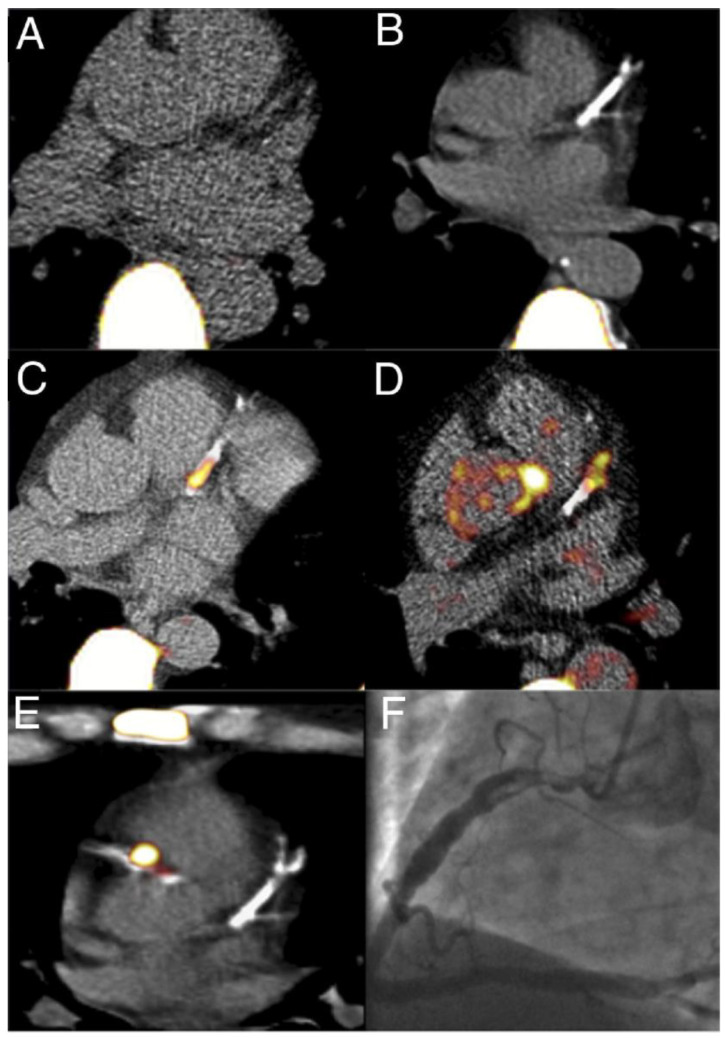
^18^F NaF PET/CT of coronary arteries demonstrating normal (no CT calcification and no PET ^18^F NaF accumulation) (**A**), macrocalcification on CT with no PET ^18^F NaF accumulation (stable plaque) (**B**), intense focal ^18^F NaF accumulation with corresponding CT calcification (**C**,**D**), intense focal accumulation of ^18^F NaF with more expansive CT calcification in a culprit lesion (**E**) with corresponding angiogram showing in situ thrombus and ulcerated plaque (**F**). Adapted from Dweck et al. [18]. 2017 Copyright the Authors.

The advancement of imaging techniques, particularly the enhanced capabilities of PET/CT using ^18^F NaF, allows for a more refined assessment of coronary arteries. This method capitalizes on detection of micro-calcifications that are often overlooked by traditional imaging modalities. As a result, it provides a significant advantage in identifying areas of potential vulnerability within arterial walls, which is crucial for understanding and managing conditions like atherosclerosis. The integration of these imaging technologies not only improves diagnostic accuracy but also enhances our understanding of the pathological processes involved in various cardiovascular diseases Figure 7.

## 2. Systematic Review of ^18^F NaF PET in Vulnerable Plaque

### 2.1. Method

A systematic review of the peer-reviewed literature associated with the use of ^18^F sodium fluoride PET specifically in coronary arteries was undertaken, and the literature has been critically evaluated to ensure relevance and reliability. Initially, the PubMed electronic database was searched using the following search terms:

- Coronary artery
- Positron emission
- Sodium fluoride

Specific search parameters restricted outputs to (inclusion criteria) the last 15 years, English language and one of the following publication types: clinical study, clinical trial, comparative study, controlled clinical trial, evaluation study, guideline, meta-analysis, observational study, randomized controlled trial, or systematic review. 

**Figure 8:**
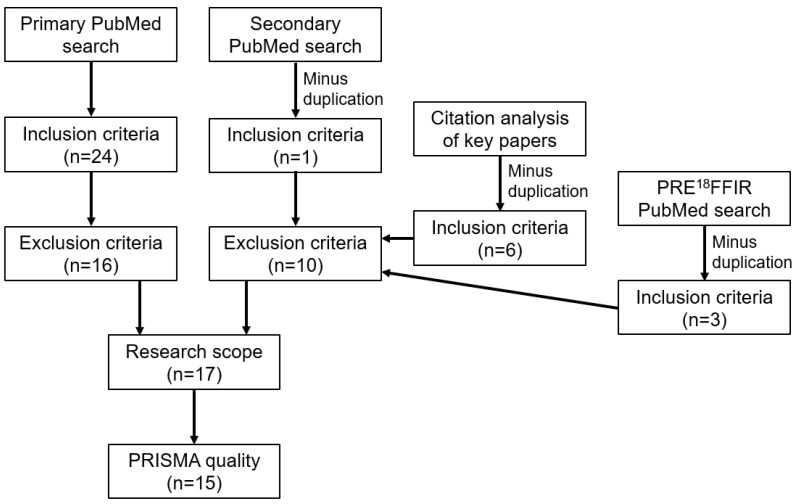
Flow chart demonstrating search criteria.

Out of 24 articles located using the primary search, nine were excluded (Figure 8) because they were either animal studies, used PET as a tool to evaluate treatment efficacy, were evaluations of instrumentation, or were evaluations of other techniques (e.g., motion correction). Manuscripts were excluded if they were editorials or letters to the editor. The marquee manuscript was identified from the PRE^18^FFIR investigators [19].

A secondary search of the PubMed electronic database was undertaken using the following search terms:

- Coronary artery
- Positron emission
- Vulnerable plaque

This search, after removal of duplications and articles outside scope, returned one additional key manuscript [20]. The “similar articles” function, “cited by” function and the citations list in PubMed were used to interrogate the two key articles [19,20] which revealed six additional manuscripts of relevance that had not already been captured. 

The PRE^18^FFIR trial (Prediction of Recurrent Events with ^18^F—Fluoride to Identify Ruptured and high-risk coronary artery plaques in patients with myocardial infarction) emerged out of the 2022 European Society of Cardiology Congress, which has investigated prediction of recurrent cardiac events using ^18^F NaF to identify vulnerable coronary artery plaques in patients with myocardial infarction. An additional search of PubMed using “PRE^18^FFIR” as the search string returned three additional manuscripts that met the inclusion criteria and which had not already been identified.

Papers were further excluded if they did not have a specific focus on ^18^F NaF PET/CT in coronary arteries. Evaluation of abstracts revealed only 17 articles that were aligned with this scope. Analysis of the papers indicated only 15 (Table 3) met the minimum standards of quality using the preferred reporting items for systematic review and meta-analysis protocol (PRISMA-P) [21]. 

### 2.2. Results

Interestingly, 13 of the articles shared common authors, which reflected involvement in the PRE^18^FFIR trial or affiliation to research conducted at Cedars Sinai Medical Centre in the USA and, therefore, a degree of homogeneity in results might be expected. Early prospective investigation of ^18^F NaF PET indicated, not surprisingly, that activity was higher in patients with atherosclerosis (*p =* 0.003), with higher rates of previous cardiac events (*p =* 0.016), presence of angina (*p =* 0.023), and also correlated with CT-CAC (r = 0.652) [18]. Soon after, a prospective clinical trial was undertaken in 37 acute MI patients with ^18^F NaF uptake (median maximum tissue to background ratio) higher (1.66) in culprit lesions than non-culprit lesions (1.24; *p* < 0.0001) [28]. By comparison, ^18^F FDG showed a ratio of 1.71 and 1.58 (*p* = 0.34) for culprit and non-culprit lesions, respectively. IVUS with features of high-risk plaques had a mean ^18^F NaF ratio of 1.90. In another early prospective investigation of ^18^F NaF PET in CAD indicated that, among 30 patients, that good agreement between observers and between patients was noted for culprit lesions [20]. 

The primary outcomes of the PRE^18^FFIR trial report in a prospective, longitudinal, international multi-center cohort study [19] investigated the association between coronary atherosclerotic plaque accumulation of ^18^F NaF PET/CT and future coronary events in patients with recent myocardial infarction (MI). The study enrolled 704 patients aged 50 years or older with recent MI (within 21 days) and multi-vessel CAD across nine centers in four countries between September 2015 and February 2020. ^18^F NaF PET and CTCA were performed in all patients. Coronary micro-calcification activity (CMA) was quantitatively assessed using FusionQuant (Cedars-Sinai Medical Center) based on volume and intensity of ^18^F NaF uptake. In the median follow-up of 4 years, cardiac death or non-fatal MI occurred in 11.2% of those with increased plaque activity (CMA > 1.56) and 6.7% among those with CMA of zero (HR, 1.82; 95% CI, 1.07–3.10; *p* = 0.03). The prognostic power of ^18^F NaF for hard events remained significant (HR, 1.76; 95% CI, 1.00–3.10; *p* = 0.05); even after adjustment for clinical characteristics, CTCA findings, GRACE score, and severity of obstructive CAD, cardiac death or non-fatal MI remained associated with high ^18^F NaF plaque activity.

Secondary analysis of PRE^18^FFIR trial data indicated that that ^18^F NaF accumulation in coronary plaque was associated with twice the risk of MI (HR = 2.1; *p* = 0.013) with increased risk associated with uptake in multiple coronary vessels (HR = 2.4; *p* = 0.002) [22]. Importantly, those treated with revascularization had a reduced risk (HE = 1.02) compared to those untreated (HR = 3.86; *p* = 0.024). An important prospective study in 101 post-coronary artery bypass graft patients with a median follow-up of 40 months revealed that ^18^F NaF accumulation in coronary arteries was predictive of perioperative MI (OR = 1.8; *p =* 0.018) [27]. A TBR_max_ greater than 3.0 had a 3.7-fold increase in perioperative MI while greater than 3.6 was associated with a 5.5-fold increased risk of major cardiovascular or cerebrovascular events.

In 32 patients with coronary atherosclerosis (111 lesions in total) assessed with CT and ^18^F NaF PET, both per patient and per lesion correlation showed that ^18^F NaF uptake was associated with plaque burden and correlated with a history of MI [32]. A related investigation used similar data (32 patients and 112 lesions) with 2-year follow-up or cardiac event [24]. While those experiencing a cardiac event were more likely to have an abnormal CT-CAC, no statistically significant correlation was noted (*p* = 0.16) while cardiac events had higher ^18^F NaF accumulation than those event-free (HR = 8.2; *p* = 0.0034). In a 5-year follow-up among 40 patients (142 lesions) with coronary atherosclerosis, ^18^F NaF accumulation using a pixel maximum to background activity radiomic value of 1.29 as the cutoff, was associated with a HR of 5.4 for major cardiac event in the next 5 years (*p =* 0.034) [25].

A contemporary prospective clinical trial enrolled 293 patients with known CAD who underwent ^18^F NaF PET/CT imaging and were followed up for a mean of 42 months [23]. ^18^F NaF accumulation (CMA) was quantitatively assessed using FusionQuant (Cedars-Sinai Medical Center). High ^18^F NaF activity (CMA > 1.56) conferred a HR 7.1 (95% CI 2.2–25.1, *p =* 0.003) for MI, outperformed all clinical and cardiac CT parameters and remained an independent predictor after adjusting for clinical risk factors, CTCA parameters, extent and severity of CAD, REACH, and SMART risk scores. ^18^F NaF PET outperformed (ROC AUC of 0.73) CT CAC risk scores (UAC = 0.54). A similar study in 136 patients (50 patients, 86 controls), the area under the curve (AUC) with receiver operator characteristic (ROC) analysis was 0.74 for ^18^F NaF PET and only 0.44 for CT CAC [26]. ^18^F NaF coronary uptake at baseline has also been reported, in 183 patients, to correlate with disease progression indicated by worsening CT-CAC scores at 12 months [31]. These findings were replicated in another study in 111 patients where a baseline ^18^F NaF CMA > 1.56 at baseline predicted progression of CT-CAC scores (*p =* 0.001) [29]. An early study compared ^18^F NaF PET to IVUS and suggested that in 14 of 15 (93.3%) of high-risk plaques identified with IVUS, had high ^18^F NaF accumulation [30]. Additionally, ^18^F NaF had a larger plaque burden than indicated by IVUS. 

Interobserver, interobserver, and interscan reproducibility was evaluated in 19 patients using ^18^F NaF PET repeated 12 days apart [33]. All values were 100% (agreement) for both the presence of uptake (CMA > 0) or absence of uptake (CMA = 0). Interobsever repeatability of CMA (mean difference of −0.02 per vessel and −0.03 per patient) was undertake with Bland Altman analysis and revealed 0.24 per vessel and 0.22 per patient, which has 95% of data points within the 95% limits of agreement (strong agreement between matched pairs). Similarly, for interobserver repeatability of CMA (mean difference of −0.01 per vessel and −0.04 per patient) was 0.30 per vessel and 0.29 per patient and 95% within the 95% limits of agreement about the −0.01 mean difference. Likewise, interscan repeatability was within the 95% limits of agreement for 95% of data points with a mean difference of 0.02 per vessel and −0.03 per patient paired with coefficient of repeatability of 0.33 and 0.32 respectively. 

## 3. Discussion 

Vulnerable plaque represents a dynamic inflammatory process that can lead to plaque rupture, making its imaging a crucial area in non-invasive diagnostic cardiology. There is an urgent need for effective early identification of vulnerable plaques, as timely treatment and management can significantly enhance plaque stability and save lives. Although techniques such as CTCA and cMRI show potential in identifying vulnerable plaques, they also have notable limitations. Traditional imaging methods that focus on the morphological characteristics of atherosclerotic plaques do not provide insights into their vulnerability. In contrast, molecular imaging with PET/CT holds significant promise for clinical applications. The molecular hallmarks of vulnerable plaques present ideal targets for imaging. However, challenges remain in visualizing these features within the small coronary arteries due to factors such as attenuation, physiological motion, residual tracer in the bloodstream, and potential myocardial accumulation of the tracer. Furthermore, the effectiveness of molecular probes developed for vulnerable plaques in carotid or aortic studies does not translate well to coronary arteries.

Currently, ^18^F NaF PET/CT imaging is one of the most widely used molecular imaging techniques for assessing plaque vulnerability [12,16]. While calcification can occur in both stable and vulnerable plaques, as indicated by CAC, ^18^F NaF tends to accumulate in micro-calcifications associated with high-risk, spotty calcification typical of vulnerable plaques rather than in macro-calcifications found in stable plaques [12,16]. This method achieves high target-to-background ratios due to rapid extraction from blood without significant myocardial accumulation of the tracer. Additionally, ^18^F NaF is easy to produce, readily available, and involves a brief imaging protocol lasting approximately 20 min. Its interpretation is intuitive, and validated quantitative analyses enhance diagnostic reliability by providing complementary confidence in image diagnosis. The short half-life of ^18^F NaF also contributes to its safety profile by minimizing radiation exposure to patients.

In contrast, while ^18^F FDG is commonly available and utilized as an inflammation marker in plaques, its performance in coronary arteries does not match that observed in carotid arteries due to issues like myocardial uptake and tracer retention in blood [12,16]. Alternative options for assessing inflammation include ^68^Ga DOTATATE or ^64^Cu SarTATE, which exploit macrophage overexpression of SSTR2 [14]. Early results from studies using these tracers in coronary arteries have shown promise and appear superior to ^18^F FDG. Among other molecular targets, ^68^Ga pentixafor, marker for chemokines, and ^68^Ga FAPI, indicating fibrous cap erosion, emerge as particularly promising candidates for stratifying vulnerable plaques due to their high target-to-background ratios [12,13,14].

Imaging vulnerable coronary plaques is a critical aspect of cardiovascular risk assessment. ^18^F FDG and ^18^F NaF PET are the two promising radionuclide agents that have clinical trial evidence for the detection of vascular plaque. Other radionuclides, while showing promise, lack clinical trial evidence and remain in an infancy state for clinical application. Of these two, clinical trial evidence on the efficacy of ^18^F FDG PET for vulnerable plaque imaging in the coronary arteries remains very limited [34,35], and ^18^F NaF PET has emerged as a superior modality compared to ^18^F FDG PET in this context [7]. The advantages of ^18^F NaF PET in coronary plaque imaging are particularly noteworthy due to the unique challenges posed by coronary artery anatomy and physiology:

- Coronary plaque specificity is demonstrated to be exceptional for detecting micro-calcification in coronary atherosclerotic plaques by ^18^F NaF, a hallmark of vulnerable lesions. This specificity arises from the ability of ^18^F NaF to reflect the exchange of hydroxyl groups in hydroxyapatite crystals, which is a crucial step in the calcification process of coronary plaques.
- Coronary imaging challenges due to their small size, constant motion, and proximity to the metabolically active myocardium is overcome by ^18^F NaF to provide:
  ◦ Low background activity due to rapid clearance of ^18^F NaF from the circulation, resulting in minimal background activity in the myocardium even after just 1 h. This allows for accurate quantification of coronary plaque uptake without complex target-to-background ratio measurements.
  ◦ Superior coronary visualization because the low myocardial uptake of ^18^F NaF enables clear visualization of coronary plaques.
  ◦ Efficient imaging protocols with ^18^F NaF require no patient preparation and short imaging protocols.
- Early detection of vulnerable coronary plaques because ^18^F NaF accumulation in micro-calcification before visibility on CT, allowing earlier intervention. Furthermore, ^18^F NaF uptake can differentiate between active and indolent calcification in coronary arteries, providing crucial information on plaque activity and potential instability.

Larger prospective studies are needed to validate the prognostic value of ^18^F NaF PET in more homogeneous and diverse patient populations. Studies should investigate whether ^18^F NaF PET can guide therapeutic decisions, such as the use of intensive lipid-lowering therapies or novel anti-inflammatory agents. Optimized imaging protocols, including delayed imaging techniques and improved image reconstruction methods, should be explored to enhance image quality and reduce radiation exposure. The combination of ^18^F NaF PET with other imaging modalities or biomarkers to improve risk stratification warrants investigation. Cost-effectiveness analyses are needed to assess the utility of ^18^F NaF PET in clinical practice for risk stratification and treatment decision-making. Long-term follow-up studies should evaluate the natural history of plaques with increased ^18^F NaF uptake and their relationship to clinical outcomes.

## 4. Conclusions

^18^F NaF PET demonstrates strong predictive ability for future myocardial infarction and cardiovascular events in patients with established CAD. It provides prognostic information independent of traditional risk factors, clinical risk scores, and measures of CAD severity. The CMA score, which measures ^18^F NaF uptake across the entire coronary vasculature, shows superior predictive performance compared to traditional methods. ^18^F NaF PET allows for non-invasive evaluation of coronary plaque activity, potentially identifying high-risk patients who may benefit from more intensive therapies. Further research is required to validate ^18^F NaF PET as a clinical tool for vulnerable plaque assessment and potentially guide personalized treatment strategies in patients with CAD.

## Figures and Tables

**Table 1:** Morphologically based characteristics for atherosclerotic plaque imaging [2,6,7,12,13].

Imaging Approach	Morphological Target	Invasiveness
CATH	Degree of stenosis, location of stenosis, number of stenoses	Invasive
CTCA	Degree of stenosis, location of stenosis, number of stenoses	Noninvasive
CAC	Atherosclerotic burden, stratification of cardiac event rate, and survival	Noninvasive
cMRI	Structural and functional changes associated with stenosis	Noninvasive
Echocardiography	Functional changes associated with stenosis	Noninvasive
Myocardial perfusion SPECT and PET	Hemodynamic significance of stenosis	Noninvasive
Intravascular ultrasound	High-resolution assessment of plaque morphology	Invasive
Optical coherence tomography	High-resolution assessment of plaque morphology	Invasive
Near-infrared spectroscopy	High-resolution assessment of plaque morphology	Invasive
^99m^Tc, ^111^In, ^123^I, ^18^F and ^68^Ga labelled LDL	Thickening of intima (fatty streak)	Noninvasive
^18^F-4V	Monocyte adhesion to VCAM-1	Noninvasive
^68^Ga fucoidan	Monocyte adhesion to P-selectin	Noninvasive
^99m^Tc or ^111^In leukocytes	Monocyte migration	Noninvasive

**Table 2:** Among the various radiotracers for molecular assessment of plaque vulnerability, only ^18^F FDG in inflammatory cells, ^68^Ga DOTATATE or ^64^Cu SarTATE in activated macrophages, ^68^Ga FAPI in the thin fibrous cap, and ^18^F NaF in micro-calcification demonstrate target-to-background ratios suitable for imaging coronary arteries [12,13,14,15,16].

Imaging Approach	Radiopharmaceutical/Probe	Molecular Target	Evidence (Coronary Arteries)
Monocyte migration	^111^In or ^99m^Tc WBCs ^68^Ga DOTA-ECL1i	Migrating leukocytes	Limited
Luminal thrombus	^111^In-platelets ^99m^Tc apcitide	Thrombus Glycoproteins activated by fibrinogen	Limited
Macrophage activity or density	^18^F FDG ^11^C Acetate or ^18^F FAC ^68^Ga DOTATATE or ^64^Cu SarTATE ^68^Ga fucoidan ^11^C choline or ^18^F fluorocholine	Glycolysis, GLUT-1 and 3 expression Lipids in macrophages SSRT_2_ Macrophage density and P-selectin adhesion Macrophage activation	Moderate Limited
Receptor expression	^68^Ga DOTATATE or ^64^CuSarTATE ^68^Ga Pentixafor Radiolabelled IL-2	SSRT_2_ Chemokine IL-2	Moderate for SSRT_2_ Mostly carotid
Neo-angiogenesis	^68^Ga-NOTA-PRGD2 ^18^F galacto-RGD ^18^F alphatide II ^18^F flotegatide ^18^F fluciclatide	Integrin α_v_β_3_ expression	Mostly carotid
Glycolysis	^18^F FDG	Inflammation	Moderate with limitations in coronaries
Calcification	^18^F NaF	Hydroxyapatite	Strong for micro-calcification
Permeability (protease)	^11^C, ^18^F, ^123^I, ^68^Ga and ^99m^Tc labelled MMPs or MMP inhibitors ^18^F or ^68^Ga FAPI	MMP FAP	Emerging
Lipid concentration	^11^C Acetate or ^18^F FAC ^99m^Tc, ^111^In, ^123^I, ^68^Ga, ^18^F, ^89^Zr LDL	Fatty streaks, macrophages, foam cells, lipid-rich core	Limited
Hypoxia	^18^F FMISO		Limited
Apoptosis	^99m^Tc Annexin ^68^Ga-Annexin V	Apoptotic macrophages and foam cells	Limited
Inflammation	^18^F FDG ^18^F-4V ^18^F Florbetaben	Endothelial activation due to inflammation Inflammation in endothelial cells and macrophages	Limited
Adhesion molecules	^18^F-4V	VCAM-1	Limited
Interleukin-2 expression	^99m^Tc-HYNIC-IL-2	Activated T-lymphocytes	Mostly carotid

**Table 3:** Summary of key literature.

Patient Number	Study Type	Outcomes	Citation
704	Prospective	^18^F NaF accumulation predicts all-cause mortality (HR = 2.43) and cardiac death or non-fatal MI (HR = 1.82),	[19]
691	Prospective	Increased CMA of ^18^F NaF has 2.1-fold increase in risk of MI over no uptake. Untreated had nearly fourfold increased risk of MI than those treated in patients with ^18^F NaF uptake.	[22]
293	Prospective	^18^F NaF coronary accumulation predicts MI and, specifically, a CMA greater than 1.56 has HR of 7.1 for future MI.	[23]
32	Prospective	^18^F NaF accumulation had HR of 8.2 for cardiac event in next 2 years.	[24]
40	Prospective	^18^F NaF accumulation with maximum TBR_max_ greater than 1.29 had a HR of 5.4 for major cardiac event in the next 5-years.	[25]
136	Retrospective	^18^F NaF uptake differentiated healthy controls from atherosclerotic patients, but CT-CAC could not.	[26]
101	Prospective	TBR_max_ > 3.0 had a 3.7-fold increase in perioperative MI while > 3.6 was associated with a 5.5-fold increased risk of major cardiovascular or cerebrovascular events.	[27]
119	Prospective	^18^F NaF activity was higher in patients with atherosclerosis (*p =* 0.003), higher rates of previous cardiac events (*p =* 0.016), angina (*p =* 0.023), and correlated with CT-CAC (r = 0.652).	[18]
37	Prospective	^18^F NaF TBR_max_ is higher (1.66) in culprit lesions than non-culprit lesions (1.24; *p* < 0.0001). By comparison, ^18^F FDG showed a ratio of 1.71 and 1.58 (*p =* 0.34) for culprit and non-culprit lesions, respectively. IVUS with features of high-risk plaques had a mean ^18^F NaF ratio of 1.90.	[28]
101	Prospective	Baseline ^18^F NaF CMA > 1.56 predicted progression of CT-CAC scores (*p =* 0.001).	[29]
51	Prospective	^18^F NaF positive lesions had high plaque burden than IVUS and OCT. 14/15 IVUS and OCT identified high-risk lesions had high ^18^F NaF uptake.	[30]
183	Prospective	CT-CAC scores increased at 12 months in those with baseline increased accumulation of ^18^F NaF.	[31]
32	Retrospective	^18^F NaF accumulation in coronary arteries correlates with a history of MI and CT-CAC.	[32]
30	Prospective	^18^F NaF uptake in culprit lesions is reproducible.	[20]
19	Prospective	100% intraobserver, interobserver, and interscan agreement for the presence (CMA > 0) or absence (CMA = 0) of coronary ^18^F NaF uptake.	[33]

CMA is coronary micro-calcification activity; HR is hazard ratio; OR is odds ratio; MI is myocardial infarction; TBR_max_ is target to blood ratio maximum; CT-CAC is CT calcium score; IVUS is intravascular ultrasound.

## Data Availability

No new data were created or analyzed in this study.
